# Hydrocephalus 2015, The 7^th^ Annual Meeting of the International Society for Hydrocephalus and CSF Disorders (ISHCSF)

**DOI:** 10.1186/2045-8118-12-S1-I1

**Published:** 2015-09-18

**Authors:** Mark Hamilton

**Affiliations:** 1 Department of Clinical Neurosciences University of Calgary, Division of Neurosurgery, Foothills Hospital, 12th Floor, 1403 - 29th Street NW, Calgary, AB Canada T2N 2T9

## 

The 7^th^ Annual Meeting of the International Society for hydrocephalus and Cerebrospinal Fluid Disorders (ISHCSF) is being held in Banff, Alberta, Canada September 19^th^-21^st^, 2015.

There are three satellite symposia accompanying the main Congress. On September 17^th^, the **1^st^ Calgary Hydrocephalus Symposium** will be hosted by the Cumming School of Medicine to provide an intimate opportunity to explore state-of-the-art hydrocephalus care. On September 18^th^, the satellite **Normal Pressure Hydrocephalus (NPH) Symposium** will be held in Banff, providing an opportunity to share and learn from the experience of international experts regarding this challenging hydrocephalus disorder. We also welcome our colleagues and members of the **International Hydrocephalus Imaging Working Group (IHIWG)**. IHIWG members include Neurologists, Neurosurgeons, Neuroradiologists and Physicists who will have their autumn meeting on September 18^th^ in Banff.

**Hydrocephalus 2015** will cover the full range of CSF disorders affecting both children and adults. The focus of work done in our field is the patient and to this end, the theme of three special sessions of this Congress will be **“The Patient”**. These special sessions will examine: 1) Quality Improvement and Safety in Hydrocephalus Care; 2) Transition and Adult Hydrocephalus Care; and 3) Ethics and Patient Involvement in Clinical Research. These sessions will provide a foundation for the research that will be presented during **Hydrocephalus 2015** which will include sessions dealing with pediatric hydrocephalus, adult hydrocephalus, advanced Neuro-imaging techniques in hydrocephalus, CSF shunt design and technology and intracranial pressure. We will also have special sessions devoted to young investigators and will recognize those with the best oral presentations and best posters.

We have a great social program to span the conference. We start with the Welcoming Reception on September 18^th^, party at a bar-be-que buffet barn dance on September 19^th^ and then celebrate at the Denim and Diamonds Gala Dinner on September 20^th^. Our very special guest speaker for the Gala Dinner is Dr. Michael Bliss, renowned historian and author of “Harvey Cushing. A Life in Surgery”.

